# Sampling a wide swathe of primate genetic diversity

**DOI:** 10.1016/j.xgen.2023.100358

**Published:** 2023-07-12

**Authors:** Evan E. Eichler

**Affiliations:** 1 Department of Genome Sciences, University of Washington School of Medicine, Seattle, WA, USA; 2 Howard Hughes Medical Institute, University of Washington, Seattle, WA 98195, USA

## Abstract

Two studies published in *Science* report the deepest survey of primate genetic diversity using short-read sequencing to sample ∼47% of extant species. Kuderna et al.^1^ investigate genetic diversity, mutation rates, and our primate phylogeny, while Gao et al.^2^ use the data to better classify disease-causing mutations.

## Teaser

Two studies published in *Science* report the deepest survey of primate genetic diversity using short-read sequencing to sample ∼47% of extant species. Kuderna et al. investigate genetic diversity, mutation rates, and our primate phylogeny, while Gao et al. use the data to better classify disease-causing mutations.

## Main text

Following the publication of the draft human genomes,^3^^,^^4^ calls were made for the reconstruction of the evolutionary history of each of the ∼3 billion base pairs of our genetic code. This was motivated by the desire to better identify the genetic differences that make us uniquely human and to improve our understanding of disease-causing mutations. Two recent papers^1^^,^^2^ make significant strides in realizing this grand challenge.

**Figure 1:**
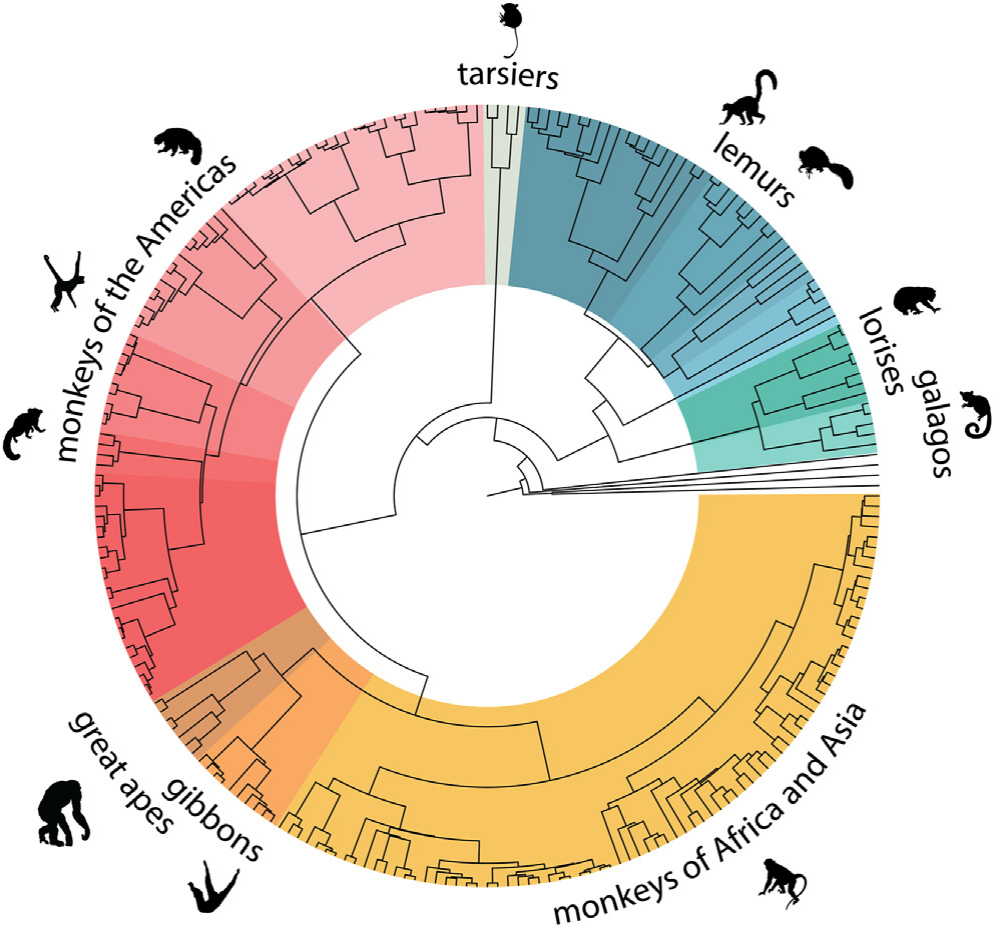
Primate sequence diversity Phylogenetic tree of 233 sequenced primate genomes (adapted from data generated by Kuderna et al.^1^)

Taking advantage of the evolutionary diversity across the primate phylogeny (Figure 1), Illumina whole-genome sequence data were generated from 703 individuals representing 211 distinct species. These data were combined with previous primate population studies^5^ to yield 809 genomes from 233 different primate species, representing approximately half of the 521 estimated extant species.

Kuderna et al.^1^ explored primate evolution, including genome-wide patterns of genetic diversity within and between primate groups. Mapping the data to 36 higher-quality reference genomes and using 27 fossil calibration points, they constructed a comprehensive primate phylogenetic tree (Figure 1). They estimated that prosimians and simians diverged 58–63 million years ago (mya). Their estimates push back the divergence of chimpanzee and human between 6.9 and 9 mya.

This dataset suggests that mutation rates vary by ∼6-fold among different primate lineages (0.25–1.62 × 10^−8^ substitutions/generation). Lemurs harbor the lowest mutation rates and great apes the highest. Humans exhibit low levels of diversity—just greater than several endangered primate species. A positive correlation between mutation rate and generation time is mostly attributed to lower mutation rates in species with larger effective population sizes. Surprisingly, 63% of the human-specific variants with high frequency in humans, yet absent in Neanderthals and Denisovans, are observed in at least one other primate species. This suggests mutational recurrence and cautions against defining lineage-specific events from examinations of limited numbers of species, although the low number of archaic genomes available should be noted. Using this broader survey of primate genomes and requiring an allele frequency of 99.9% in humans, they highlight 124 missense *Homo sapiens* mutations for more in-depth functional characterization.

Gao and colleagues^2^ focused specifically on protein-coding variation. They use primate variation data to better distinguish benign from disease-causing protein-altering mutations. The close genetic relationship among primates means that the majority of genes and gene models can be easily tracked across species. Each branch in the primate phylogeny allows for the accumulation of potentially benign missense mutations—essentially multiple runs of natural selection. This increases the power to detect naturally occurring protein-coding changes as additional primate phylogenetic branches are surveyed. Thus, the authors created a primate population variant database of 4.3 million common missense variants—50 times larger than the clinical variant database (ClinVar) generated from human data.

Utilizing ClinVar annotations, 98.7% of these common primate protein-altering variants are estimated to be benign. Armed with this knowledge, Gao et al. train a semi-supervised 3D convolutional neural network, PrimateAI-3D, which incorporates predicted alpha-2-fold protein structure and the distribution of common missense variants from the primate multiple sequence alignment to better predict benign and pathogenic variants. PrimateAI-3D outperforms 15 machine-learning classifiers and estimates an increase in pathogenic variant yield discovery from 1.36- to 2.0-fold, depending on the thresholds applied. Such gains are important for disorders like neurodevelopmental delay and autism, where *de novo* missense mutations are still mostly classified as variants of unknown significance though more likely to contribute to a greater proportion of patients than loss-of-function mutations.^6^

While the two studies make important contributions, several limitations and future directions are acknowledged by the authors. The analyses were based on short reads, and gene characterization was limited to genes with unambiguous 1:1 mapping between human and nonhuman primates. There has been considerable duplication during the evolution of the primate lineage—including biomedically relevant genes and regions implicated in unique features of human brain development.^7^^,^^8^ While unclear how many human protein-coding genes were excluded, it is indicated that ∼20% of the genome (0.6 Gbp) could not be considered in this analysis. Related to this, the use of both incomplete references from human (GRCh38) and nonhuman primates may have introduced errors resulting from incorrect sequence data comparisons and mapping. Given the modest number of primate species, a complete telomere-to-telomere genome using long-read sequencing data from each primate species will be a critical step in understanding the evolution of all genes.^9^^,^^10^

Also, for most species, they sequenced only a single representative (133/233) with uncertain disease status. The average of 3.5 individuals per species is skewed by earlier studies whose data were included, such as the deeply surveyed ape lineages.^5^ Sequencing multiple individuals should provide greater clarity on common versus rare species-specific variants and improve the predictive value of their algorithm. Finally, many more functional tests of the predictions of PrimateAI-3D, including comparisons with deep mutational scanning data and functional assays in human tissues, are needed. Differences in selective pressure among even closely related species may affect what mutations are tolerated.

In summary, both papers make a compelling case of how more complete genetic information across primates improves our understanding of human genetic variation, including functional changes unique to our species. For disease gene discovery, the sequencing of additional primate genomes complements large-scale surveys of human population controls because of the relatively low degree of genetic diversity within our species. These analyses are particularly timely given that 60% of primates are endangered. There is an irony in that the genetic information present in nonhuman primates—facing extinction due to human interference—may improve our understanding of our own species and the health of our children.
